# Protocol for developing an evidence-based expert consensus on efficacy evaluation and comorbidity management of integrated Chinese and Western medicine for pediatric tic disorders: a GRADE-based modified Delphi study

**DOI:** 10.3389/fneur.2026.1891900

**Published:** 2026-08-21

**Authors:** Mengmeng Bao, Yaru Wang, Ning Zhang, Jiaqi Wang, Ji Li

**Affiliations:** 1 Department of Pediatrics, Guang’anmen Hospital, China Academy of Chinese Medical Sciences, Beijing, China; 2 Pingyang Hospital of Traditional Chinese Medicine, Zhejiang University of Traditional Chinese Medicine, Zhejiang, China; 3 Handan Integrated Traditional Chinese and Western Medicine Hospital, Handan, Hebei, China

**Keywords:** consensus development, Delphi, GRADE, integrative medicine, protocol, tic disorders

## Abstract

**Background:**

Pediatric tic disorders (TD) are common neurodevelopmental conditions frequently complicated by attention-deficit/hyperactivity disorder, obsessive-compulsive disorder, anxiety, and depression. Integrated Chinese and Western medicine is widely used in China, but its efficacy evaluation and comorbidity management lack standardized, evidence-anchored consensus methodologies.

**Objective:**

To describe the protocol for developing an evidence-based expert consensus on efficacy evaluation and comorbidity management of integrated Chinese-Western medicine for pediatric TD, using a GRADE-informed modified Delphi process.

**Methods:**

The consensus will be developed in six phases: (1) setup and scoping, including a multidisciplinary, cross-regional Development Panel and identification of core clinical questions; (2) systematic literature search, quality appraisal (AMSTAR 2, RoB 2, ROBINS-I, QUADAS-2, AGREE II, and JBI checklists), and GRADE certainty assessment; (3) a three-round modified Delphi survey with ≥75% consensus threshold, anonymous voting, and controlled feedback; (4) a planned hybrid consensus meeting to adjudicate unresolved items, with two-stage voting (adoption then strength) and predefined decision rules; (5) drafting, external review, and final endorsement; and (6) dissemination and updating. Good Practice Statements (GPS) will be distinguished from evidence-based recommendations.

**Expected outcomes:**

This protocol seeks to generate: (1) core recommendation statements; (2) an integrated efficacy evaluation framework spanning tic severity, TCM syndromes, and functional status; (3) targeted management pathways for prevalent comorbidities (ADHD, OCD, anxiety/depression); and (4) standardized safety monitoring principles.

**Conclusion:**

This protocol presents a transparent, reproducible, and GRADE-aligned methodology for developing an integrative consensus in pediatric TD. It may serve as a methodological reference for future integrative guideline development.

## 1 Introduction

Tic disorders (TD) are childhood-onset neurodevelopmental disorders characterized by recurrent motor and/or vocal tics (1–3). The condition is frequently comorbid with other psychiatric and behavioral disorders, including attention-deficit/hyperactivity disorder (ADHD), obsessive-compulsive disorder (OCD), anxiety disorder (AD), and depressive disorder (DD), which significantly affect patients’ learning ability, social functioning, and mental health (4). Current epidemiological surveys indicate that the global prevalence of TD is 1–3% (5–7). Among patients with Tourette syndrome (TS), the comorbidity rate with ADHD ranges from 50 to 60%, and with OCD from 40 to 60%. The comorbidity rates for DD and AD are 36.4 and 53.5%, respectively, imposing a considerable burden on society (8–12).

Although evidence-based behavioral and pharmacological treatments are available, the clinical management of TD remains challenging (13). Fundamentally, symptom profiles are inherently heterogeneous and fluctuate over time (14). Compounding this clinical variability, comorbidity patterns differ considerably and frequently demand individualized prioritization in treatment planning (15). Recent studies have begun to explore neurodevelopmental markers that may contribute to understanding the biological heterogeneity of TD and inform future personalized assessment strategies (13, 16). Beyond tic suppression alone, clinical management should consider broader outcomes, including cognitive functioning, emotional well-being, school performance, family burden, and long-term safety (17). Collectively, these complexities indicate that a symptom-centered outcome model alone is insufficient for comprehensive TD management.

In China, integrated Chinese and Western medicine has been increasingly applied in pediatric TD management (18, 19). In practice, Western medicine may provide relatively standardized diagnostic frameworks, validated symptom measures, and pharmacological or behavioral interventions, whereas Chinese medicine may contribute syndrome-based pattern recognition, individualized herbal strategies, and a broader emphasis on systemic regulation. Clinicians frequently combine these approaches in an attempt to improve tic control, reduce comorbid symptoms, enhance overall functioning, and mitigate treatment-related adverse effects (20). However, despite its growing use, integrative management remains methodologically understandardized (21).

Several notable gaps appear to persist in the field. Primarily, a standardized efficacy evaluation system for integrated pediatric TD management has yet to be fully established, while inconsistent TCM syndrome differentiation can potentially constrain cross-study reproducibility (19, 20). Furthermore, comorbidity management tends to remain fragmented, frequently lacking a cohesive framework that synthesizes diverse clinical outcomes. Finally, previous consensus documents may occasionally lack optimal methodological transparency, particularly regarding evidence evaluation and the adjudication of expert divergence (22, 23).

In contexts of heterogeneous evidence and complex clinical practice, consensus credibility depends fundamentally on methodological transparency. A prospectively registered protocol mitigates post-hoc bias, clarifies decision rules, and enhances overall reproducibility (24). Therefore, this study outlines a predefined, GRADE-informed, modified Delphi framework to develop an evidence-based consensus on the integrative management of pediatric TD. Specifically, our objectives are to: (1) establish a multidisciplinary, cross-regional expert panel to identify priority clinical questions; (2) systematically synthesize available evidence and determine certainty using the GRADE approach; (3) adjudicate recommendations through iterative Delphi surveys and a structured consensus meeting; and (4) finalize a standardized, actionable framework for efficacy evaluation and comorbidity management.

## 2 Methods

### 2.1 Study design and overview

The protocol for this expert consensus was prospectively registered on the PREPARE (Practice Guideline REgistration for transPAREncy) platform (PREPARE-2026CN750) (25, 26). To ensure methodological transparency and academic rigor, the development and reporting of this protocol strictly adhere to the Preferred Reporting Items for Systematic Reviews and Meta-Analysis Protocols (PRISMA-P) statement (27) and A Reporting Tool for Practice Guidelines in Health Care (RIGHT) statement (28).

**Figure 1:**
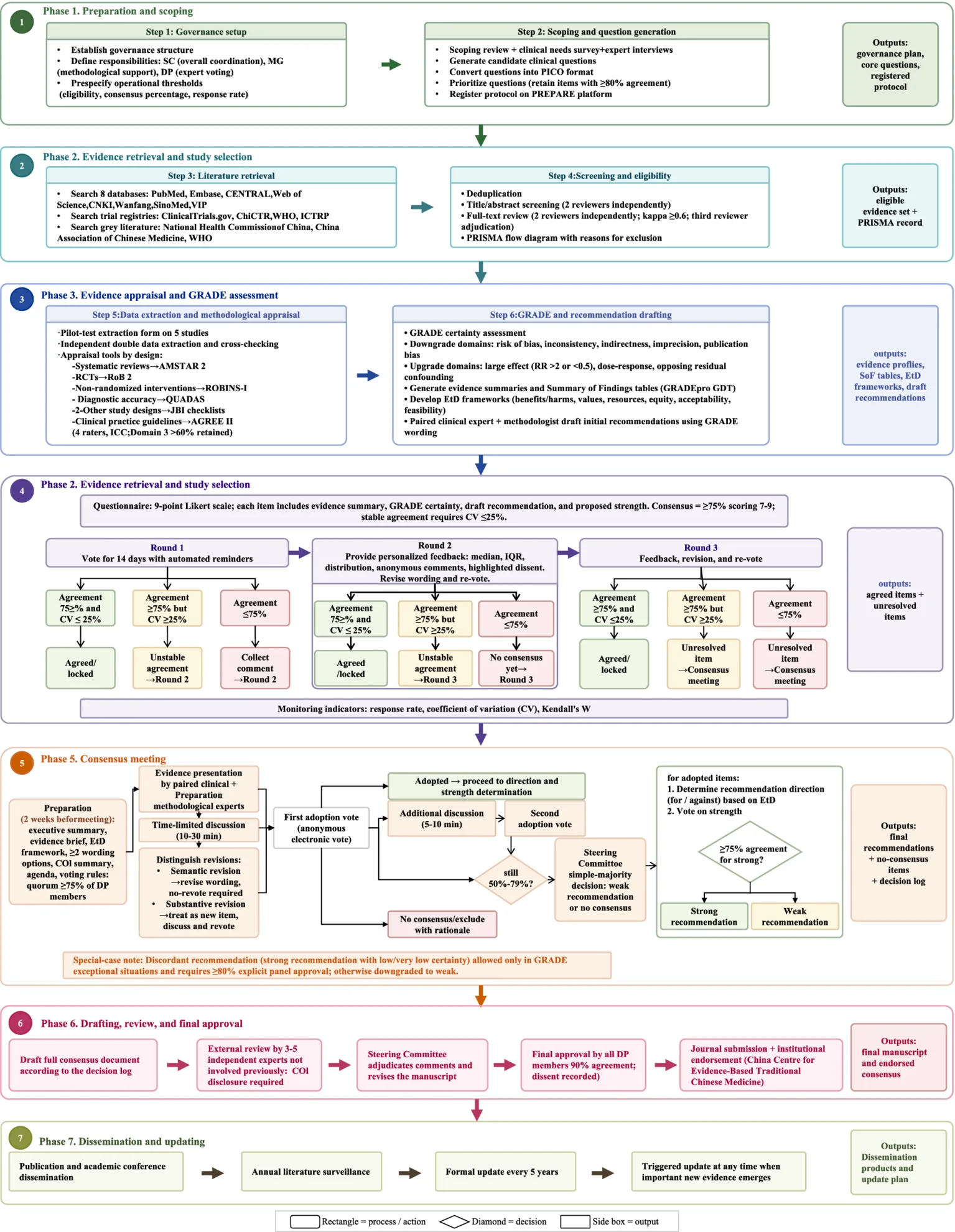
Guideline development flowchart.

This study employs an evidence-based, modified Delphi consensus design integrated with the Grading of Recommendations Assessment, Development, and Evaluation (GRADE) approach (24, 29). The protocol was designed to maximize methodological transparency, reproducibility, and explicit linkage between evidence certainty and recommendation strength. A full workflow diagram showing phases, steps, actions, and study status will be provided in Figure 1.

The overall consensus development process is systematically delineated into six distinct phases: (1) setup and scoping to define the target population, comorbidities, and core clinical questions; (2) systematic evidence identification, quality appraisal, and GRADE certainty assessment; (3) a three-round modified Delphi survey to establish preliminary consensus on draft recommendations; (4) a planned formal consensus meeting to resolve contentious items and finalize recommendation strengths; (5) drafting and multi-level internal/external review of the consensus document; and (6) final dissemination and updating strategy.

As of the time of protocol submission, Phases 1–3 had been completed, while Phases 4–6 remained pending. Detailed study status is reported in Section 3.2.

### 2.2 Phase 1: setup and scoping

#### 2.2.1 Governance structure

The consensus is governed by a Steering Committee, a Development Panel, and a Methodology and Evidence Support Group (23, 24, 30). The Steering Committee is responsible for overall coordination, methodological oversight, progress monitoring, document harmonization, dispute adjudication, and version control. The Development Panel provides disciplinary expertise, participates in question refinement, Delphi voting, and final decision-making, and contributes to drafting the final consensus document. The Methodology and Evidence Support Group provides assistance with literature retrieval, evidence synthesis, methodological standardization, and GRADE judgment.

#### 2.2.2 Steering committee

The Steering Committee comprises protocol authors who were responsible for the conception, methodological design, coordination, and implementation of the consensus project, together with clinical experts in pediatric tic disorders, a specialist in integrative medicine, and an evidence-based medicine methodologist. In addition to their governance roles, the committee is tasked with organizing panel meetings, managing the distribution and data collection of the Delphi surveys, and maintaining all administrative records throughout the consensus process.

#### 2.2.3 Development panel

Panelists were selected to ensure comprehensive representation across pediatric traditional Chinese medicine (TCM), general pediatrics, pediatric neurology, child psychiatry and psychology, integrative medicine, and evidence-based medicine (23, 30, 31). Geographic diversity was explicitly prioritized during the recruitment process to mitigate local practice bias and enhance the national applicability of the final recommendations. Panel eligibility criteria and specific operational thresholds were prespecified by the Steering Committee in alignment with established consensus guidelines: (1) holding a chief physician title or above, or an equivalent senior academic rank; (2) possessing a minimum of 10 years of relevant clinical, academic, or methodological experience; (3) demonstrating substantive clinical or methodological expertise in pediatric tic disorders, closely related neurodevelopmental or psychiatric conditions, integrative medicine, traditional Chinese medicine pediatrics, or evidence-based guideline development; and (4) demonstrating peer-recognized expertise in the relevant field. Peer-recognized expertise was defined as meeting at least one of the following criteria: invited lectures, oral presentations, or session chairing at national or provincial academic conferences; membership in relevant academic societies, guideline panels, or consensus groups; participation in major clinical research, guideline, or consensus projects; or publication as first author, corresponding author, or senior author of at least one peer-reviewed article related to tic disorders, pediatric neurodevelopmental or psychiatric disorders, integrative medicine, traditional Chinese medicine pediatrics, or evidence-based methodology.

#### 2.2.4 Methodology and evidence support

The methodology and evidence support component includes experts in evidence-based medicine and information retrieval. This group contributed to the design of the literature search strategy, evidence extraction templates, appraisal procedures, GRADE judgments, and the analytic framework used to summarize Delphi responses. Methodological support also helped ensure that recommendation wording and certainty grading were aligned.

#### 2.2.5 Conflict of interest management

A standardized conflict of interest (COI) protocol is implemented prior to the initiation of the study. All participants are required to complete a formal declaration detailing any financial, academic, or institutional relationships relevant to the consensus topics. The Steering Committee reviews all submitted forms. If a potential COI is identified, the involved member is recused from voting on or formulating recommendations related to that specific conflict. COI declarations are documented and will be updated prior to the formal consensus meeting.

#### 2.2.6 Scope of the consensus

During the scoping phase, the applicability and scope of the consensus were preliminarily defined. The target population was specified as children and adolescents younger than 18 years diagnosed with TD, encompassing both uncomplicated cases and those with major comorbidities (specifically ADHD, OCD, anxiety, and depressive disorders). The preliminary objectives of the consensus are threefold: (1) to establish a standardized efficacy evaluation framework for integrated Chinese and Western medicine in pediatric TD; (2) to develop comorbidity-specific management pathways; and (3) to define key safety monitoring and management principles relevant to integrative care. Furthermore, the anticipated end-users of the finalized consensus were primarily identified as pediatricians, pediatric neurologists, child psychiatrists, TCM pediatricians, and clinicians practicing in integrative medicine settings. These preliminary scoping decisions will be subject to formal discussion and approval during the subsequent modified Delphi survey and consensus conference.

#### 2.2.7 Identification and refinement of clinical questions

##### 2.2.7.1 Action 1: generation of candidate questions

An initial pool of clinical questions was gathered through three parallel channels: a scoping review of existing TD guidelines and recent literature, semi-structured expert interviews, and a clinical needs assessment evaluating the real-world challenges faced by frontline pediatricians and TCM practitioners.

##### 2.2.7.2 Action 2: PICO formulation

The Steering Committee consolidated the candidate questions, removing duplicates and translating the actionable items into the structured PICO format. The methodological framework was defined as follows: Population (P) comprised children and adolescents under 18 years of age with a confirmed clinical diagnosis of TD, including those with primary comorbidities (ADHD, OCD, anxiety, or depression); Intervention (I) involved integrative Chinese and Western medicine approaches; Comparator (C) included conventional Western monotherapy, standard behavioral interventions, baseline care, or no intervention; and Outcomes (O) encompassed tic symptom reduction, comorbidity improvement, TCM syndrome score changes, functional quality of life, and safety metrics.

##### 2.2.7.3 Action 3: prioritization and finalization

The structured PICO questions were presented to the Development Panel. The panel evaluated the clinical relevance, urgency, and feasibility of each item using a Likert scale. Questions meeting a predefined consensus threshold (≥80% agreement) were retained as the core clinical questions to guide the subsequent evidence synthesis and recommendation formulation.

### 2.3 Phase 2: evidence identification and appraisal

#### 2.3.1 Literature search

We will conduct systematic searches of PubMed/MEDLINE, Embase, the Cochrane Central Register of Controlled Trials (CENTRAL), Web of Science, and four primary Chinese databases (CNKI, Wanfang Data, SinoMed, and VIP). Ongoing and unpublished studies will be identified via ClinicalTrials.gov, ChiCTR, and WHO ICTRP. Grey literature will be searched via the websites of the National Health Commission of China, China Association of Chinese Medicine, WHO, and Google Scholar.

The search period will be from database inception to 31 May 2025 for efficacy and safety evidence; for epidemiological and guideline evidence, the period will be restricted to the past 10 years. No language restrictions will be applied. Search strategies will be structured around the PICO framework, using controlled vocabulary and free-text keywords (full strategies in Appendices). Each strategy will be peer-reviewed using the PRESS 2015 checklist by an independent medical librarian (32).

All searches will be re-run within 6 months before manuscript submission. Reference tracking of included studies and relevant reviews will be performed. Reporting will follow PRISMA 2020 (33) and PRISMA-Search extensions (34).

#### 2.3.2 Eligibility criteria and study selection

Eligibility criteria for evidence synthesis were defined according to the PICO framework for each clinical question (as detailed in Section 2.2.7). Exclusion criteria will include: (1) non-original research including editorials, narrative reviews, commentaries unless they are systematic reviews that provide direct evidence; (2) animal or *in vitro* studies; (3) conference abstracts without full-text availability; (4) preprints not yet peer-reviewed; and (5) studies with duplicate or clearly erroneous data.

Following the database search, all retrieved records will be imported into EndNote (version 20) for duplicate removal, then exported to Rayyan for blinded independent screening. Two independent reviewers will screen titles and abstracts, followed by full-text screening against the PICO criteria. Inter-rater reliability will be interpreted using the Landis–Koch criteria. Values below acceptable agreement (*κ* < 0.6) will trigger discrepancy review and resolution by consensus or third-party adjudication (35). Any unresolved disagreements will be adjudicated by a third senior methodologist.

The complete study selection process, including reasons for full-text exclusion, will be documented in a PRISMA flow diagram.

#### 2.3.3 Data extraction

To ensure data integrity and consistency, a standardized, structured data extraction form was developed. Prior to formal utilization, this instrument was rigorously pilot-tested on a purposive sample of five representative publications (comprising two randomized controlled trials, one cohort study, one systematic review, and one study specific to Traditional Chinese Medicine syndrome differentiation). Upon refinement and formal approval by the Steering Committee, the finalized form was deployed for independent data extraction by two methodological reviewers. Following independent extraction, the dual datasets were cross-verified. Any identified discrepancies were resolved through consensus discussion or formal adjudication by a third senior methodologist (Supplementary material).

#### 2.3.4 Quality appraisal

The methodological quality of all included literature was independently appraised by two reviewers, with inter-rater reliability quantified using Cohen’s kappa coefficient (36). Any persistent discrepancies were resolved through formal adjudication by a third senior methodologist.

For systematic reviews, methodological quality was appraised using the AMSTAR 2 instrument (37). Randomized controlled trials were assessed using the Cochrane Risk of Bias 2 (RoB 2) tool (38). Non-randomized studies of interventions, such as cohort studies and quasi-experimental studies, were evaluated utilizing the ROBINS-I tool (39). Diagnostic test accuracy studies were appraised using the QUADAS-2 instrument (40).

For study designs not covered by the instruments above, the corresponding Joanna Briggs Institute (JBI) critical appraisal checklists were applied to the remaining study designs, specifically qualitative research, prevalence studies, economic evaluations, case reports, case–control studies, and analytical cross-sectional studies (41).

Clinical practice guidelines (CPGs) were evaluated using the AGREE II instrument (42). To ensure methodological rigor, this appraisal was conducted independently by four reviewers, with inter-rater reliability calculated via the Intraclass Correlation Coefficient (ICC) (43). ICC values will be interpreted according to established criteria, with ICC ≥ 0.75 considered indicative of acceptable agreement (44). If insufficient agreement occurs, discrepancies will be reviewed and resolved through discussion among reviewers. A CPG was retained for evidence extraction only if Domain 3 (Rigor of Development) achieved a score exceeding 60%.

Crucially, all identified primary studies and systematic reviews were retained regardless of their initial quality scores. These detailed appraisals directly informed the subsequent GRADE certainty judgments, specifically guiding potential downgrading within the risk of bias domain (45).

#### 2.3.5 Evidence certainty assessment

The certainty of evidence for each clinical question was assessed using the GRADE approach. Assessments were performed independently by two methodologists who had completed formal GRADE training (29, 45–48). Disagreements were resolved through discussion or adjudication by a third GRADE methodologist from the Steering Committee. GRADEpro GDT was used to generate evidence profiles and Summary of Findings tables (49).

GRADE domains for downgrading: risk of bias, inconsistency, indirectness, imprecision, publication bias (46, 49). Domains for upgrading: large magnitude of effect, dose–response gradient, and residual confounding that would increase confidence in the observed effect (47). Upgrading was applied only to observational evidence and required explicit thresholds: large effect = RR (relative risk) > 2 or < 0.5 (based on two or more studies); very large effect = RR > 5 or < 0.2 (further upgrade). Dose–response gradient and negative bias were applied when clearly demonstrated (47). Final certainty ratings: High, Moderate, Low, Very Low (29, 45–47).

Non-clinical evidence encompassing patient values, preferences, intervention feasibility, and resource utilization will be qualitatively summarized. For qualitative evidence, GRADE-CERQual (Confidence in Evidence from Reviews of Qualitative research) will be applied where feasible, following the GRADE-CERQual Guideline (50, 51). For diagnostic test accuracy, the GRADE for DTA framework will be used (46, 48). These non-clinical assessments will be documented within the Evidence-to-Decision (EtD) frameworks but will not receive a formal GRADE certainty grade. For each draft recommendation, the rationale for upgrading or downgrading will be documented in evidence summary tables. The complete GRADE evidence profiles will be provided in Supplementary material.

#### 2.3.6 Development of evidence summaries and draft recommendation statements

The Methodology and Evidence Support Group developed standardized evidence summaries and EtD frameworks for each core clinical question (52). The EtD frameworks explicitly incorporated evidence certainty, the balance of benefits and harms, patient preferences, and clinical feasibility. Based on these frameworks, paired subgroups, comprising one clinical expert and one methodologist, drafted the preliminary recommendation statements. The wording of each statement was strictly calibrated to reflect the corresponding GRADE certainty and recommendation strength.

Crucially, for clinical questions lacking direct empirical evidence two distinct methodological pathways are established. Where a statement is considered necessary, actionable, supported by compelling indirect evidence, and judged to have unequivocal net benefit, it will be formulated as a Good Practice Statement (GPS) (53). Conversely, where the balance of effects remains uncertain due to low or very low-certainty evidence without meeting GPS criteria, the statement will be drafted as a weak recommendation (46). GPS issuance requires ≥80% panel agreement and will be labeled in the final consensus.

### 2.4 Phase 3: three-round modified Delphi survey

#### 2.4.1 Anonymity in the modified Delphi survey

During all three Delphi rounds, full anonymity was maintained among panelists: no panelist could identify the responses, comments, or identities of other panelists at any point during data collection. However, the Steering Committee had access to individual responses for the purpose of generating personalized feedback reports and managing non-response. Upon publication of the final consensus, the full panel membership will be disclosed in the acknowledgments, but individual responses will remain permanently anonymized.

#### 2.4.2 Questionnaire design

All Delphi items were derived directly from the PICO-formulated clinical questions (Section 2.2.7) and the draft recommendations generated by the paired expert-methodologist subgroups (Section 2.3.6). To provide comprehensive context, each survey item included the core clinical question, the corresponding evidence summary, the proposed GRADE certainty rating, the draft recommendation, and the suggested recommendation strength.

Expert agreement was systematically quantified using a 9-point Likert scale (1 = strongly disagree, 9 = strongly agree). To guide the iterative revision of contentious statements, panelists assigning a score of ≤6 were required to provide explicit rationales or wording modifications via a mandatory free-text field. Prior to formal deployment, the instrument was pilot-tested by three methodologists and two independent clinicians to optimize clarity and functionality. The finalized survey was administered via a secure, web-based platform to facilitate asynchronous participation, protect expert anonymity, and guarantee data integrity.

#### 2.4.3 Delphi procedures

The iterative voting process was conducted over three sequential rounds. The multidisciplinary Development Panel operated as a single, unified cohort rather than being divided into discipline-specific subpanels (54). All voting members met the predefined eligibility criteria (Section 2.2.3) and adhered to strict conflict of interest recusal protocols for specific items (Section 2.2.5). Each voting round spanned 14 days, separated by a 7 to 10-day inter-round interval for data analysis, feedback generation, and statement revision. The *a priori* consensus threshold for all rounds was defined as ≥75% agreement. This threshold was selected based on Delphi studies, which identified percentage agreement as the most frequently used consensus criterion and reported 75% as the median threshold across studies (54, 55).

Round 1: The initial questionnaire was distributed with a two-week completion window, utilizing automated reminders to maximize response rates.

Round 2: Items achieving the ≥75% consensus threshold were locked as “agreed.” For unresolved items, panelists received individualized feedback reports comparing their original scores against overall group statistics (frequency distribution, median, and interquartile range). These reports also included an anonymized summary of free-text rationales, specifically highlighting perspectives diverging from the majority view. Based on this feedback, contentious statements were systematically revised and redistributed for re-evaluation.

Round 3: This feedback-and-revision cycle was repeated. Any statements persistently failing to reach consensus after three rounds were deferred to the planned face-to-face meeting (Phase 4) for final adjudication.

#### 2.4.4 Statistical analysis of Delphi responses

Statistical analyses of the Delphi responses were performed using (SPSS version 26.0). The response, rate derived from the questionnaire recovery and effective response rates, was calculated to reflect expert engagement. At the item level, formal consensus was determined using a rigorous dual statistical criterion. The primary criterion required ≥75% of panelists to score 7 or 9 on the 9-point Likert scale. As a secondary stability indicator, the coefficient of variation (CV) was used as a secondary indicator to evaluate response dispersion and stability across items. Items meeting the agreement threshold and showing a CV ≤ 25% were classified as achieving stable consensus. The CV threshold was selected based on previous Delphi studies, where values ≤25% have been commonly used to indicate acceptable convergence of expert opinions (54). Crucially, items achieving the ≥75% agreement threshold but exhibiting a CV > 25% were flagged for further deliberation at the consensus meeting. Furthermore, descriptive measures of central tendency (median) and dispersion (interquartile range) were examined, and Kendall’s coefficient of concordance (Kendall’s *W*) was calculated to assess the overall coordination of expert opinions within each round. The stability of responses across successive Delphi rounds was additionally evaluated by comparing changes in median scores, interquartile ranges, and the proportion of panelists reaching consensus for each item. Statistical significance was set at *p* < 0.05. The detailed calculation formulas and complete item-level results will be reported in the Supplementary materials.

#### 2.4.5 Quality assurance in the Delphi process

Building upon the specific quality control measures embedded within the preceding Delphi procedures (Sections 2.4.1–2.4.4), overall methodological transparency is further guaranteed through standardized reporting. The execution and reporting of the Delphi component will strictly adhere to the ACCORD (Accurate Consensus Reporting Document) guidelines (22, 23) and the DCAT (Delphi Critical Appraisal Tool) quality standards (56). The completed DCAT checklist is provided in Supplementary material.

#### 2.4.6 Panelist retention and attrition management

Panelist retention was monitored across all three Delphi rounds and the following strategies were employed: (1) personalized email invitations from a known contact on the Steering Committee; (2) automated reminders at day 7 and day 12 for non-responders; (3) clear communication of the expected time commitment (approximately 30–45 min per round) and project timeline at the outset; (4) acknowledgment of panelists in the final publication; and (5) minimizing response burden by locking agreed items from subsequent rounds.

Participation rates for each round will be reported as the positive coefficient (Ca = completed/distributed × 100%). Attrition by disciplinary subgroup will be examined to assess whether dropout introduced systematic bias in the panel composition. To assess potential non-response and attrition bias, available characteristics of responders and non-responders will be compared descriptively across Delphi rounds. Early and late responders will also be compared in terms of agreement proportions and item-level score distributions where data permit. The primary analysis will use complete-case analysis, defined as responses from panelists who complete all three Delphi rounds, excluding those with missing item-level responses.

Panelists who completed fewer than 80% of items in a given round were excluded from that round’s analysis. Responses from panelists who participated in earlier rounds but dropped out of later rounds were not carried forward. The minimum acceptable response rate for proceeding with a subsequent round was set at 70%, consistent with published recommendations (57).

If the response rate in any round falls below 70%, the Steering Committee will implement prespecified mitigation measures before proceeding, including extending the response window by 7–14 days, sending additional personalized reminders, contacting non-responders to identify logistical barriers. If the response rate remains below 70% after these measures, the Steering Committee will compare available characteristics of responders and non-responders, examine early versus late responders descriptively, and determine whether the round should be repeated once or reported as a protocol deviation. Any persistent low response rate, differential attrition, or evidence of potential non-response bias will be transparently reported and considered when interpreting the stability and credibility of the Delphi findings.

Sensitivity analyses will be conducted using three complementary approaches to examine the robustness of consensus findings under different missing-data assumptions: (1) available-case analysis, including all valid item-level responses without imputation; (2) complete-case analysis, restricted to panelists who complete all three Delphi rounds; and (3) a conservative scenario in which all missing responses are treated as non-agreement for the purpose of calculating percentage agreement. If the consensus classification of any item differs between the primary complete-case analysis and either sensitivity scenario, the item will be flagged for further discussion at the consensus meeting and reported transparently in the final consensus document (58).

### 2.5 Phase 4: planned consensus meeting

Following completion of the three-round modified Delphi survey, a structured consensus meeting will be conducted as the final consensus step to adjudicate unresolved items, refine wording, and confirm the direction and strength of recommendations instead of reopening all Delphi-agreed items (59).

Unresolved items to be brought forward for discussion will include: (1) items that failed to achieve the pre-specified Delphi agreement threshold (≥75%); (2) items with unstable agreement across rounds with a drop of >15 percentage points in agreement or a change in the median category; and (3) items meeting the numerical threshold but accompanied by substantial qualitative concerns regarding interpretation, feasibility, or wording.

#### 2.5.1 Preparation for the meeting

The Steering Committee will be responsible for organizing and logistically coordinating the consensus meeting. The meeting will convene the members of the Steering Committee and the Development Panel. The meeting will be conducted in a hybrid format in-person attendance for Steering Committee members and local panelists, with remote participation via a secure video-conferencing platform for others. If in-person gathering becomes impossible, a fully virtual meeting will be held as a contingency; any such change will be documented as a protocol amendment.

A quorum of ≥75% of Development Panel members will be required for formal voting. The chairperson will be a methodologist with no voting rights, trained in facilitating consensus meetings to ensure neutrality and balanced participation.

To facilitate informed deliberation, a comprehensive set of preparatory materials will be distributed to all participants at least 2 weeks prior to the meeting. These materials will include: (1) executive summary of all approved and unresolved recommendations; (2) full evidence profiles and Summary of Findings tables generated by GRADEpro; (3) complete EtD frameworks for each unresolved recommendation, including panel comments from Delphi Round 3; (4) alternative wording at least two options proposed by the drafting subgroup for each unresolved item; (5) consolidated anonymous rationales from dissenting Delphi responses; (6) de-identified COI declaration summary for all attendees; (7) meeting agenda with time allocation; (8) operational voting rules and decision flowchart. Panelists will be asked to confirm receipt and review of materials at least 3 days prior to the meeting.

#### 2.5.2 Meeting procedures

The meeting will be facilitated by a non-voting moderator to ensure balanced and efficient deliberation. Steering Committee members concurrently serving on the Development Panel retain full voting rights, whereas Methodology Group members will participate strictly as non-voting technical advisors.

For each unresolved item, paired clinical and methodological leads will jointly present the evidence profiles and specific Delphi divergences, initiating a time-boxed deliberation focused on integrative applicability and feasibility. Concluding the discussion, each item will be assigned a definitive action trajectory: retain as drafted, merge with another item, split into independent statements, convert to GPS, defer due to insufficient evidence, exclude, or undergo wording revision (53). For items requiring revision, real-time modifications will be strictly categorized. Minor editorial refinements will be integrated immediately. Conversely, substantive alterations modifying a statement’s scope or direction will be designated as novel items; in accordance with ACCORD guidelines, such material changes mandate an additional deliberation cycle and a subsequent re-vote prior to finalization (22, 23). Following this rigorous adjudication process, the formalized two-stage voting protocol will be executed for all active recommendations.

#### 2.5.3 Decision rules

Voting will be conducted anonymously via an electronic audience-response system Mentimeter (60, 61). The denominator for each vote will be the number of eligible voting panelists present at the time of voting, minus those recusing themselves for conflicts of interest on that specific item. Abstentions will be excluded from the denominator. For each unresolved recommendation, a two-stage voting procedure will be applied:

##### 2.5.3.1 Step 1: adoption

For each unresolved item, a first vote will determine whether the recommendation is adopted at all:

- ≥80% agreement → adopted.
- 50–79% agreement → the item is discussed again (5–10 min) followed by a second adoption vote; if still 50–79% after second vote, the Steering Committee decides by simple majority whether to adopt as weak or defer to “no consensus.”
- <50% agreement → recorded as “no consensus” (excluded from final consensus, with reasons documented).

##### 2.5.3.2 Step 2: strength and direction

For adopted recommendations, the direction (for/against) and strength (strong/weak) are derived from the EtD framework, which explicitly considers: certainty of evidence, balance of benefits and harms, patient values and preferences, resource use, equity, acceptability, and feasibility (29, 62).

##### 2.5.3.3 Step 3: strength vote

After the EtD framework is reviewed, panelists vote on the proposed strength. A recommendation is classified as strong if ≥75% of voting panelists agree; Otherwise weak.

For the formulation of discordant recommendations defined as strong recommendations predicated on low or very low-certainty evidence will be heavily restricted. Such instances must be strictly justified by at least one of the five paradigmatic situations outlined by the GRADE Working Group and mandate a specific ≥80% panel endorsement of that exact justification (46, 63).

All votes, rationales, wording revisions, and dissenting opinions will be recorded in real time by a dedicated secretariat. A full item-by-item voting log will be published as an online supplement.

#### 2.5.4 Documentation

To maintain a rigorous methodological audit trail and ensure strict compliance with ACCORD reporting standards, a dedicated secretariat will prospectively generate five prespecified reportable outputs during and immediately following the consensus meeting. These encompass: (1) a formalized attendance and quorum record; (2) an item-by-item presentation sheet; (3) a qualitative summary of critical discussion points; (4) a comprehensive version history tracking all wording modifications and their explicit rationales; and (5) a finalized master decision log capturing the definitive status, exact vote distributions, and strength justifications for all clinical questions (Supplementary material).

Draft outputs will be distributed to all attendees within 14 days post-meeting for formal verification, allocating a 7-day window for experts to submit revision comments. Upon panel validation, these records will serve as the definitive foundation for drafting the final manuscript. Furthermore, any subsequent methodological deviations from this prespecified protocol will be systematically documented with explicit justifications and transparently reported in the final publication.

### 2.6 Phase 5: drafting, review, and endorsement

#### 2.6.1 Drafting of the consensus document

Following the consensus meeting, the Steering Committee and specifically assigned members of the Methodology Group will draft the full consensus document. The manuscript will systematically synthesize the approved recommendation statements, the corresponding evidence profiles, the comorbidity management pathways, and the specific clinical algorithms for integrative care. To ensure absolute fidelity to the experts’ decisions, the draft will be rigorously cross-checked against the master decision log and the finalized GRADE certainty ratings by the secretariat.

#### 2.6.2 External review

A formal external review process will be conducted (30, 42). The draft manuscript will be distributed to a selected group of three to five independent clinical and methodological experts. Crucially, these external reviewers must not have participated in the Delphi survey or the consensus meeting. External reviewers will be required to disclose any potential conflicts of interest prior to receiving the draft (64). They will evaluate the document using a structured appraisal form. The Steering Committee will systematically review and adjudicate all external feedback and implement necessary revisions.

#### 2.6.3 Final approval and endorsement

The revised manuscript will subsequently be circulated to all members of the Development Panel for final verification and approval. Once ≥90% approval is achieved, the document will be prepared for journal submission (65). Any dissenting opinions will be documented and reported in the final consensus file. The finalized consensus will also be submitted to China Center for Evidence-Based Traditional Chinese Medicine for formal institutional endorsement.

### 2.7 Phase 6: dissemination and updating

The finalized consensus document will be submitted for peer-reviewed publication and broadly disseminated through international academic conferences and relevant professional networks. To facilitate rapid clinical implementation, a concise, practice-oriented summary will also be developed specifically for frontline healthcare practitioners.

To maintain the currency and validity of the consensus, the Steering Committee will conduct annual surveillance of newly published literature. A formal comprehensive update is planned within 5 years, although an accelerated revision process will be initiated earlier if pivotal new clinical data or significant safety signals emerge.

## 3 Ethics and study status

### 3.1 Ethical considerations

This study methodology is fundamentally based on the systematic synthesis of previously published scientific literature and the structured aggregation of professional opinions from clinical experts. All clinical and methodological experts participating in the modified Delphi survey were fully informed of the study objectives, the data management protocols, and the intended publication plans. The voluntary completion and submission of the Delphi questionnaires by the panelists constituted their formal informed consent to participate in this consensus project.

The funding organizations had no role in the design of the Delphi questionnaire, the selection of panelists, the analysis of responses, or the formulation of recommendations. No endorsing organization participated in the voting process.

### 3.2 Current study status

**Figure 2:**
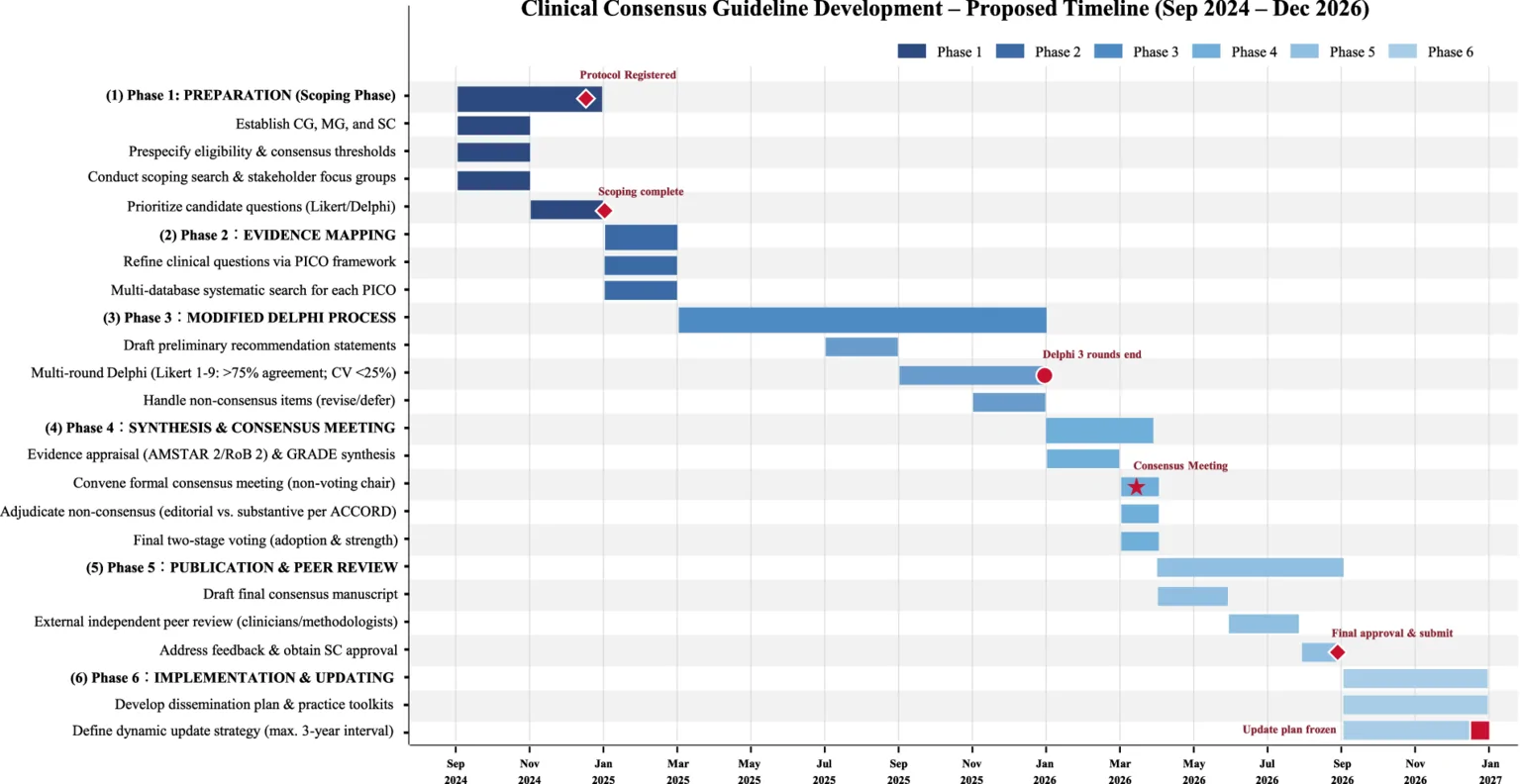
Project timeline for the pediatric TD consensus.

As of the submission of this protocol, the project has successfully completed its first three methodological phases. The initial scoping, systematic literature retrieval, data extraction, and GRADE certainty assessments have been fully conducted. Furthermore, the three-round modified Delphi survey has concluded, yielding significant expert agreement on the majority of the draft recommendation statements. The study is currently in the preparatory stage for the formal consensus meeting (Phase 4), which is scheduled to take place (Figure 2).

## 4 Discussion

This protocol addresses an methodological and clinical gap in the management of pediatric tic disorders within the context of integrated Chinese and Western medicine. Although integrative approaches are widely used in practice, their evaluation remains insufficiently standardized, particularly with respect to efficacy assessment, comorbidity management, and safety monitoring. In areas where evidence is heterogeneous and clinical decision-making extends beyond tic reduction alone, a formal consensus process is often necessary. The present protocol therefore provides a transparent, prespecified pathway for developing recommendations.

This study is methodologically anchored in an evidence-informed design. By combining systematic literature retrieval, study appraisal, and GRADE-based certainty assessment with a modified Delphi process, the protocol seeks to ensure that expert judgment is anchored to explicit evidence summaries rather than implicit preference alone (29, 54, 63). This is particularly relevant in pediatric TD, where the evidence base is uneven across clinical questions and where recommendation development requires balancing symptom control, comorbidity burden, functional outcomes, and safety (14). The multidisciplinary and cross-regional composition of the expert panel is another important strength, as it broadens the interpretive base of the consensus and may reduce single-discipline or single-center bias [(30, 31), p. 2]. In addition, the use of three Delphi rounds followed by a structured consensus meeting allows the process to combine the advantages of anonymous iterative feedback with a final stage of focused adjudication for unresolved items. The prespecification of governance, voting rules, conflict-of-interest management, and documentation procedures is also expected to improve transparency, reproducibility, and the interpretability of the final consensus (64).

Foremost, the certainty of the available evidence is likely to vary substantially across the included clinical questions, inevitably forcing some recommendations to rely on lower-certainty or indirect data. Compounding this challenge, syndrome differentiation in traditional Chinese medicine remains inherently interpretive, potentially restricting diagnostic consistency across clinicians and institutions even when a shared framework is proposed (66–69). Furthermore, regarding external validity, although the multidisciplinary panel is geographically diverse within China, its predominantly domestic composition may limit the international applicability of the resulting recommendations. Differences in healthcare systems, clinical practice patterns, cultural perceptions of traditional Chinese medicine, and the availability of integrative healthcare services may influence how these recommendations are interpreted and implemented in other settings. These considerations highlight the need for future validation and adaptation of the consensus framework in different healthcare contexts. In addition, the literature search yielded predominantly Chinese-language publications, which reflects the current landscape of integrative medicine research but may introduce potential language and publication biases. Ultimately, the consensus methodology itself harbors inescapable constraints: while Delphi rounds mitigate overt interpersonal influence, iterative feedback can subtly shape subsequent judgments, and the final non-anonymous meeting introduces group dynamics where dominance effects cannot be entirely eliminated (70).

The final consensus aims to generate four categories of outputs: (1) recommendation statements for core clinical questions related to the efficacy evaluation and comorbidity management of integrated Chinese and Western medicine in pediatric TD; (2) an integrated efficacy evaluation framework incorporating tic severity, syndrome-based assessment, and functional outcomes; (3) to develop condition-specific management pathways for major comorbidity patterns, including ADHD, OCD, and anxiety/depressive symptoms; and (4) to define safety monitoring and management principles for integrative treatment. Based on these intended outputs, this protocol hopes to contribute to the development of a more transparent and methodologically robust consensus. If successfully implemented, the resulting consensus could potentially assist in standardizing efficacy evaluation, facilitating the integration of comorbidity assessment into routine decision-making, and offering a clearer framework for future clinical research in pediatric TD.

## 5 Conclusion

This protocol describes a process for developing an evidence-based expert consensus on efficacy evaluation and comorbidity management of integrated Chinese and Western medicine for pediatric tic disorders. By combining systematic evidence appraisal, explicit GRADE judgments, a three-round modified Delphi process, and a planned formal consensus meeting, the study aims to produce a trustworthy and clinically usable consensus framework. The protocol may also serve as a methodological reference for future consensus development in integrative pediatric medicine.
