# Supplementary material for: Protocol for developing an evidence-based expert consensus on efficacy evaluation and comorbidity management of integrated Chinese and Western medicine for pediatric tic disorders: a GRADE-based modified Delphi study
Source: Front Neurol. 2026 Aug 21;17:1891900. doi: 10.3389/fneur.2026.1891900 (PMC13541544; doi:10.3389/fneur.2026.1891900)
Supplement: Supplementary file 1 [file Supplementary_file_1.docx]

**Supplementary Materials**

| **Table of Contents** | |
| --- | --- |
| Appendix 1: | Consensus Development Reporting Checklist |
| Appendix 2: | Expert Conflict of Interest Declaration Form |
| Appendix 3: | Expert Panel Characteristics |
| Appendix 4: | Evolution of Clinical Questions |
| Appendix 5: | Complete Search Strategies by Database |
| Appendix 6: | Foundational Data Extraction Form |
| Appendix 7: | GRADE Evidence Assessment Table |
| Appendix 8: | Delphi Questionnaire Sample |
| Appendix 9: | Expert Engagement and Concordance Coefficients |
| Appendix 10: | Structured External Review Form |

# Appendix 1: Protocol reporting checklist

| Section | Item | Description | Location in Protocol | Status |
| --- | --- | --- | --- | --- |
| 1. Basic information and registration | 1a | Title identifies the document as a protocol for consensus/guideline development | Title page | Reported |
|  | 1b | Registration platform and registration number | Section 2.1 | Reported |
|  | 1c | Year/version/update status of the protocol | Title page / Section 2.1 | Reported |
|  | 1d | Funding source(s) and role of the funder | Funding statement / Acknowledgments / Section 3.1 | Reported or to be added at submission* |
| 2. Scope, objectives, and target users | 2a | Overall objective(s) of the consensus | Introduction / Section 1 | Reported |
|  | 2b | Target population: age, diagnosis, and comorbidities | Section 2.2.6 | Reported |
|  | 2c | Target users: specialties and clinical settings | Section 2.2.6 | Reported |
|  | 2d | Scope of the consensus: efficacy evaluation, comorbidity management, safety | Section 2.2.6 | Reported |
|  | 2e | Core clinical questions formulated using PICO | Section 2.2.7 / Appendix 4 | Reported |
| 3. Governance, stakeholder involvement, and COI | 3a | Governance structure: Steering Committee, Development Panel, Methodology and Evidence Support Group | Section 2.2.1 | Reported |
|  | 3b | Steering Committee composition and responsibilities | Section 2.2.2 | Reported |
|  | 3c | Development Panel composition: disciplines and geographic distribution | Section 2.2.3 / Appendix 3 | Reported |
|  | 3d | Methodology and Evidence Support Group composition and role | Section 2.2.4 | Reported |
|  | 3e | Conflict of interest disclosure and management procedures | Section 2.2.5 / Appendix 2 | Reported |
|  | 3f | Patient/public involvement | Not included | Not applicable |
| 4. Evidence retrieval and study selection | 4a | Databases and information sources searched | Section 2.3.1 | Reported |
|  | 4b | Search period and date of last search | Section 2.3.1 | Reported |
|  | 4c | Full search strategies for each database | Appendix 5 | Reported |
|  | 4d | Inclusion and exclusion criteria | Section 2.3.2 | Reported |
|  | 4e | Study selection procedures, duplicate screening, and adjudication process | Section 2.3.2 | Reported |
|  | 4f | PRISMA flow diagram | Appendix 6 | Reported |
| 5. Evidence appraisal and certainty assessment | 5a | Data extraction procedures and standardized extraction form | Section 2.3.3 / Appendix 7 | Reported |
|  | 5b | Study quality appraisal tools by design | Section 2.3.4 | Reported |
|  | 5c | GRADE certainty assessment domains for downgrading and upgrading | Section 2.3.5 / Appendix 8 | Reported |
|  | 5d | GRADE evidence profiles and Summary of Findings tables | Section 2.3.5 / Appendix 8 | Reported |
|  | 5e | Evidence-to-Decision (EtD) frameworks | Section 2.3.6 | Reported |
| 6. Consensus methodology: Delphi design and analysis | 6a | Type of consensus method used (GRADE-informed modified Delphi) | Section 2.1 / Section 2.4 | Reported |
|  | 6b | Anonymity procedures during Delphi rounds | Section 2.4.1 | Reported |
|  | 6c | Questionnaire design and pilot testing | Section 2.4.2 / Appendix 9 | Reported |
|  | 6d | Number of Delphi rounds and inter-round feedback procedures | Section 2.4.3 | Reported |
|  | 6e | Consensus threshold(s) and scoring approach | Section 2.4.3 / Section 2.4.4 | Reported |
|  | 6f | Statistical analysis: response rate, median, IQR, CV, Kendall’s W | Section 2.4.4 / Appendix 10 | Reported |
|  | 6g | Delphi quality assurance measures | Section 2.4.5 | Reported |
|  | 6h | Retention and attrition management | Section 2.4.6 | Reported |
| 7. Consensus meeting and decision rules | 7a | Objective of the consensus meeting as the final adjudication step | Section 2.5 | Prespecified |
|  | 7b | Eligibility of items carried forward to the meeting | Section 2.5 | Prespecified |
|  | 7c | Meeting preparation, agenda, and pre-circulated materials | Section 2.5.1 | Prespecified |
|  | 7d | Quorum requirement and voting eligibility | Section 2.5.1 / Section 2.5.3 | Prespecified |
|  | 7e | Meeting procedures, including discussion, revision, and re-voting | Section 2.5.2 | Prespecified |
|  | 7f | Decision rules for adoption, strength, deferral, or exclusion | Section 2.5.3 | Prespecified |
|  | 7g | Documentation, audit trail, and protocol deviation reporting | Section 2.5.4 | Prespecified |
| 8. Recommendation formulation | 8a | Draft recommendation wording linked to GRADE certainty and EtD considerations | Section 2.3.6 | Reported |
|  | 8b | Criteria for recommendation direction and strength | Section 2.5.3 | Prespecified |
|  | 8c | Good Practice Statements distinguished from evidence-based recommendations | Section 2.3.6 / Section 2.5.2 | Reported |
|  | 8d | Handling of substantively revised items or unresolved items | Section 2.5.2 / Section 2.5.3 | Prespecified |
| 9. Review, endorsement, and approval | 9a | External review by independent experts | Section 2.6.2 | Prespecified |
|  | 9b | Conflict of interest disclosure for external reviewers | Section 2.6.2 | Prespecified |
|  | 9c | Final panel approval threshold | Section 2.6.3 | Prespecified |
|  | 9d | Institutional endorsement process | Section 2.6.3 | Prespecified |
| 10. Ethics, dissemination, updating, and study status | 10a | Ethical considerations and expert consent | Section 3.1 | Reported |
|  | 10b | Current study status and completed phases | Section 3.2 | Reported |
|  | 10c | Publication and dissemination plan | Section 2.7 | Planned |
|  | 10d | Updating strategy and surveillance cycle | Section 2.7 | Planned |

# Appendix 2. Expert Conflict of Interest Disclosure and Management Form

All members of the Steering Committee, Development Panel, Methodology and Evidence Support Group, and external reviewers are required to complete this form before participating in the consensus process. Disclosures will be reviewed by the Steering Committee, and management actions, including recusal from discussion or voting on specific items, will be documented in accordance with established principles for disclosure and management of interests in guideline and consensus development.

## Expert Conflict of Interest (COI) Disclosure Form

**Full name:** ___________________________________________

**Institution / affiliation:** _________________________________

**Department:** __________________________________________

**Professional title:** ______________________________________

**Specialty area:** __________________________________________

**Role in this project:** _____________________________________

**Date of declaration:** _____________________________________

### **Reporting period**

Please disclose all relevant interests that are **current** or occurred within the **past 36 months**, as well as any ongoing relationships that could reasonably be perceived as influencing your judgment^[1]^.

| Category | Description | Disclosure (Yes/No) | Details (if Yes) | Time period | Steering Committee management decision* |
| --- | --- | --- | --- | --- | --- |
| 1. Financial interests | Research grants, consulting fees, advisory board fees, honoraria, speaker fees, travel support, stock ownership, patent royalties, or other financial relationships related to tic disorders, traditional Chinese medicine, psychotropic medications, behavioral interventions, or relevant diagnostic/assessment tools |  |  |  |  |
| 2. Academic interests | Authorship of potentially competing guidelines or consensus statements, strongly stated prior publications or public positions on the consensus topics, editorial roles, or academic advancement directly linked to the recommendations |  |  |  |  |
| 3. Institutional interests | Institutional affiliations, policies, partnerships, or commercial collaborations that could influence recommendations or implementation of the consensus |  |  |  |  |
| 4. Clinical practice interests | Significant revenue, referral patterns, or service-line dependence arising from clinical activities directly addressed by this consensus |  |  |  |  |
| 5. Personal / professional relationships | Close professional, supervisory, mentoring, or collaborative relationships that could reasonably affect independent judgment on relevant recommendations |  |  |  |  |
| 6. Other interests | Any other financial or non-financial interests that could be perceived as influencing your judgment |  |  |  |  |

* Possible management decisions: **No action required / Partial recusal from discussion / Recusal from voting on specific item(s) / Full recusal from relevant section(s) / Other (specify).**

### **Additional information**

If you answered “Yes” to any category above, please specify:

1. the nature of the relationship or activity;
2. the organization or entity involved;
3. whether the interest is current, recent, or ongoing; and
4. which topic(s) or recommendation area(s) may be affected.

### **Declaration statement**

I declare that the information provided in this form is complete and accurate to the best of my knowledge. I understand that disclosed interests will be reviewed by the Steering Committee and may result in management actions, including recusal from discussion or voting on specific recommendations where appropriate. I also agree to update this declaration promptly if any relevant interests arise or change during the consensus development process^[1,2]^.

Signature: ________________________	Date: ________________________

**For Steering Committee use only**

Reviewed by: __________________________________________

Date of review: ________________________________________

Management decision: ___________________________________

Items/topics affected (if applicable): _______________________

[1]	Schünemann HJ, Al-Ansary LA, Forland F, Kersten S, Komulainen J, Kopp IB, Macbeth F, Phillips SM, Robbins C, van der Wees P, Qaseem A, for the Board of Trustees of the Guidelines International Network. Guidelines International Network: Principles for Disclosure of Interests and Management of Conflicts in Guidelines. Ann Intern Med, 2015, 163(7)：548-553.

[2]	Norris SL, Holmer HK, Ogden LA, Burda BU. Conflict of interest in clinical practice guideline development: a systematic review. PloS one, 2011, 6(10)：e25153.

# Appendix 3. Expert panel characteristics

This appendix summarizes the composition of the expert panel involved in the development of the consensus, including disciplinary background, geographic distribution, professional rank, project role, and conflict-of-interest disclosure status. All members met the predefined eligibility criteria described in Section 2.2.3.

**Table A3-1. Development Panel: Composition by Discipline, Region, and Title**

| No. | Name | Institution | Province/Region | Discipline | Area of expertise | Professional Title | Role | COI Declared (Yes/No) | COI Management Action (if applicable) |
| --- | --- | --- | --- | --- | --- | --- | --- | --- | --- |
| 1 |  |  |  |  |  |  |  |  |  |
| 2 |  |  |  |  |  |  |  |  |  |
| 3 |  |  |  |  |  |  |  |  |  |
| … |  |  |  |  |  |  |  |  |  |

SC = Steering Committee; DP = Development Panel. All experts met the eligibility criteria defined in Section 2.2.3.

**Table A3-2. Summary of Panel Composition**

| **Characteristic** | **n (%)** |
| --- | --- |
| **Total panel members** |  |
| Discipline | |
| TCM Pediatrics |  |
| General Pediatrics |  |
| Pediatric Neurology |  |
| Child Psychiatry/Psychology |  |
| Integrative Medicine |  |
| Evidence-Based Medicine |  |
| Professional Title | |
| Chief Physician |  |
| Professor |  |
| Associate Professor |  |
| Geographic Regions Represented | |
| East China |  |
| North China |  |
| South China |  |
| Central China |  |
| Southwest China |  |
| Northwest China |  |
| Northeast China |  |

# Appendix 4. Evolution of Clinical Questions

This appendix documents how candidate questions were generated, refined, converted to PICO format, and prioritized.

| No. | Source | Candidate question | Action taken / rationale | PICO-formulated version | Final status | Mapped final question |
| --- | --- | --- | --- | --- | --- | --- |
| 1 | Scoping review / expert interview / needs assessment | [Original wording] | [Reason for refinement] | [Population–Intervention–Comparator–Outcomes] | Retained / Merged / Excluded | [Final core question ID] |
| 2 | Scoping review / expert interview / needs assessment | [Original wording] | [Reason for refinement] | [PICO] | Retained / Merged / Excluded | [Final core question ID] |

Appendix 5: Complete Search Strategies by Database

All searches were conducted from database inception to May 2025. The following strategies were peer-reviewed by an independent information specialist.

# A5-1. PubMed/MEDLINE search strategy

## A5-1a. Intervention / efficacy / safety evidence

| **Step** | **Search terms** |
| --- | --- |
| #1 | ("Tic Disorders"[Mesh] OR "Tourette Syndrome"[Mesh] OR tic disorder*[tiab] OR Tourette*[tiab] OR motor tic*[tiab] OR vocal tic*[tiab] OR chronic tic*[tiab]) |
| #2 | ("Child"[Mesh] OR "Adolescent"[Mesh] OR "Infant"[Mesh] OR pediatric*[tiab] OR paediatric*[tiab] OR child*[tiab] OR adolescen*[tiab] OR youth[tiab] OR infant*[tiab]) |
| #3 | ("Integrative Medicine"[Mesh] OR "Medicine, Chinese Traditional"[Mesh] OR "Drugs, Chinese Herbal"[Mesh] OR integrative medicine[tiab] OR integrated Chinese and Western[tiab] OR traditional Chinese medicine[tiab] OR Chinese medicine[tiab] OR Chinese herbal*[tiab] OR herbal medicine[tiab] OR TCM[tiab] OR zhongyi[tiab] OR "zhong xi yi"[tiab] OR combined therapy[tiab] OR integrat*[tiab] OR acupuncture[tiab]) |
| #4 | (efficacy[tiab] OR effectiveness[tiab] OR outcome*[tiab] OR response[tiab] OR evaluation[tiab] OR assess*[tiab] OR safety[tiab] OR "adverse event*"[tiab] OR "adverse effect*"[tiab] OR tolerability[tiab]) |
| #5 | #1 AND#2 AND#3 |
| #6 | #1 AND#2 AND#3 AND #4 |
| Final | #5 OR #6 |

## A5-1b. Comorbidity evidence

| **Step** | **Search terms** |
| --- | --- |
| #1 | ("Tic Disorders"[Mesh] OR "Tourette Syndrome"[Mesh] OR tic disorder*[tiab] OR Tourette*[tiab] OR motor tic*[tiab] OR vocal tic*[tiab] OR chronic tic*[tiab]) |
| #2 | ("Child"[Mesh] OR "Adolescent"[Mesh] OR "Infant"[Mesh] OR pediatric*[tiab] OR paediatric*[tiab] OR child*[tiab] OR adolescen*[tiab]) |
| #3 | ("Attention Deficit Disorder with Hyperactivity"[Mesh] OR "Obsessive-Compulsive Disorder"[Mesh] OR "Anxiety Disorders"[Mesh] OR "Depressive Disorder"[Mesh] OR ADHD[tiab] OR obsessive-compulsive[tiab] OR OCD[tiab] OR anxiety[tiab] OR depression[tiab] OR depressive[tiab] OR comorbid*[tiab]) |
| Final | #1 AND#2 AND#3 |

## A5-1c. Epidemiology / guideline evidence

| **Step** | **Search terms** |
| --- | --- |
| #1 | ("Tic Disorders"[Mesh] OR "Tourette Syndrome"[Mesh] OR tic disorder*[tiab] OR Tourette*[tiab]) |
| #2 | ("Child"[Mesh] OR "Adolescent"[Mesh] OR pediatric*[tiab] OR paediatric*[tiab] OR child*[tiab] OR adolescen*[tiab]) |
| #3 | (epidemiolog*[tiab] OR prevalence[tiab] OR incidence[tiab] OR guideline*[tiab] OR consensus[tiab] OR recommendation*[tiab]) |
| Final | #1 AND#2 AND#3 AND ("2015/01/01"[Date - Publication] : "2025/05/31"[Date - Publication]) |

# A5-2. Embase (via Ovid) search strategy

## A5-2a. Intervention / efficacy / safety evidence

| **Step** | **Search terms** |
| --- | --- |
| 1 | 'tic disorder'/exp OR 'gilles de la tourette syndrome'/exp OR 'tic disorder*':ti,ab OR tourette*:ti,ab OR 'motor tic*':ti,ab OR 'vocal tic*':ti,ab OR 'chronic tic*':ti,ab |
| 2 | 'child'/exp OR 'adolescent'/exp OR 'infant'/exp OR pediatric*:ti,ab OR paediatric*:ti,ab OR child*:ti,ab OR adolescen*:ti,ab OR youth:ti,ab |
| 3 | 'integrative medicine'/exp OR 'traditional chinese medicine'/exp OR 'chinese herbal drug'/exp OR 'acupuncture'/exp OR 'traditional chinese medicine':ti,ab OR 'chinese medicine':ti,ab OR 'chinese herbal*':ti,ab OR TCM:ti,ab OR 'integrated chinese and western':ti,ab OR integrat*:ti,ab OR acupuncture:ti,ab |
| 4 | efficacy:ti,ab OR effectiveness:ti,ab OR outcome*:ti,ab OR response:ti,ab OR evaluation:ti,ab OR assess*:ti,ab OR safety:ti,ab OR 'adverse event*':ti,ab OR 'adverse effect*':ti,ab OR tolerability:ti,ab |
| 5 | 1 AND 2 AND 3 |
| 6 | 1 AND 2 AND 3 AND 4 |
| Final | 5 OR 6 |

## A5-2b. Comorbidity evidence

| **Step** | **Search terms** |
| --- | --- |
| 1 | 'tic disorder'/exp OR 'gilles de la tourette syndrome'/exp OR 'tic disorder*':ti,ab OR tourette*:ti,ab |
| 2 | 'child'/exp OR 'adolescent'/exp OR child*:ti,ab OR adolescen*:ti,ab OR pediatric*:ti,ab OR paediatric*:ti,ab |
| 3 | 'attention deficit hyperactivity disorder'/exp OR 'obsessive compulsive disorder'/exp OR 'anxiety disorder'/exp OR 'depressive disorder'/exp OR ADHD:ti,ab OR OCD:ti,ab OR anxiety:ti,ab OR depression:ti,ab OR depressive:ti,ab OR comorbid*:ti,ab |
| Final | 1 AND 2 AND 3 |

## A5-2c. Epidemiology / guideline evidence

| **Step** | **Search terms** |
| --- | --- |
| 1 | 'tic disorder'/exp OR 'gilles de la tourette syndrome'/exp OR 'tic disorder*':ti,ab OR tourette*:ti,ab |
| 2 | 'child'/exp OR 'adolescent'/exp OR child*:ti,ab OR adolescen*:ti,ab OR pediatric*:ti,ab OR paediatric*:ti,ab |
| 3 | epidemiolog*:ti,ab OR prevalence:ti,ab OR incidence:ti,ab OR guideline*:ti,ab OR consensus:ti,ab OR recommendation*:ti,ab |
| Final | 1 AND 2 AND 3; limited to 2015-2025 |

# A5-3. Cochrane Central Register of Controlled Trials (CENTRAL)

## A5-3. CENTRAL search

| **Step** | **Search terms** |
| --- | --- |
| #1 | [mh "Tic Disorders"] OR [mh "Tourette Syndrome"] OR "tic disorder*" OR Tourette* OR "motor tic*" OR "vocal tic*" OR "chronic tic*" |
| #2 | [mh Child] OR [mh Adolescent] OR [mh Infant] OR pediatric* OR paediatric* OR child* OR adolescen* |
| #3 | [mh "Integrative Medicine"] OR [mh "Medicine, Chinese Traditional"] OR [mh "Drugs, Chinese Herbal"] OR [mh Acupuncture] OR "traditional Chinese medicine" OR "Chinese medicine" OR "Chinese herbal*" OR TCM OR "integrated Chinese and Western" OR integrat* OR acupuncture |
| #4 | efficacy OR effectiveness OR outcome* OR evaluation OR safety OR "adverse event*" OR "adverse effect*" |
| Final | #1 AND#2 AND#3 |

# A5-4. Web of Science Core Collection search strategy

## A5-4a. Intervention / efficacy / safety evidence

| **Step** | **Search terms** |
| --- | --- |
| #1 | TS=("tic disorder*" OR "Tourette syndrome" OR Tourette* OR "motor tic*" OR "vocal tic*" OR "chronic tic*") |
| #2 | TS=(pediatric* OR paediatric* OR child* OR adolescen* OR youth OR infant*) |
| #3 | TS=("integrative medicine" OR "traditional Chinese medicine" OR "Chinese medicine" OR "Chinese herbal*" OR TCM OR acupuncture OR "integrated Chinese and Western" OR integrat*) |
| #4 | TS=(efficacy OR effectiveness OR outcome* OR response OR evaluation OR assess* OR safety OR "adverse event*" OR "adverse effect*" OR tolerability) |
| Final | #1 AND#2 AND#3 |

## A5-4b. Comorbidity evidence

| **Step** | **Search terms** |
| --- | --- |
| #1 | TS= ("tic disorder*" OR "Tourette syndrome" OR Tourette*) |
| #2 | TS=(pediatric* OR paediatric* OR child* OR adolescen*) |
| #3 | TS= (ADHD OR "attention deficit" OR OCD OR "obsessive-compulsive" OR anxiety OR depression OR depressive OR comorbid*) |
| Final | #1 AND#2 AND#3 |

## A5-4c. Epidemiology / guideline evidence

| **Step** | **Search terms** |
| --- | --- |
| Final | TS= ("tic disorder*" OR "Tourette syndrome" OR Tourette*) AND TS= (child* OR adolescen* OR pediatric* OR paediatric*) AND TS= (epidemiolog* OR prevalence OR incidence OR guideline* OR consensus OR recommendation*) Timespan: 2015-01-01 to 2025-05-31 |

# A5-5. CNKI search strategy

## A5-5a. Intervention / efficacy evidence

| **Step** | **Search terms** |
| --- | --- |
| #1 | SU=('抽动障碍' OR '妥瑞综合征' OR '抽动-秽语综合征' OR '抽动秽语综合征' OR '多发性抽动' OR '慢性抽动' OR '一过性抽动' OR '抽动症') |
| #2 | SU=('儿童' OR '青少年' OR '小儿' OR '学龄儿童' OR '未成年人' OR '儿科') |
| #3 | SU=('中西医结合' OR '中西医' OR '中西医联合' OR '中西药' OR '中医治疗' OR '中西医结合疗法' OR '中医药' OR '中药' OR '中药方剂' OR '中成药' OR '中药制剂' OR '针刺' OR '针灸') |
| Final | #1 AND#2 AND#3 |

## A5-5b. Safety evidence

| **Step** | **Search terms** |
| --- | --- |
| #1 | 同上 |
| #2 | 同上 |
| #3 | 同上 |
| #4 | SU=('安全性' OR '不良反应' OR '不良事件' OR '耐受性') |
| Final | #1 AND#2 AND#3 AND #4 |

## A5-5c. Comorbidity evidence

| **Step** | **Search terms** |
| --- | --- |
| #1 | 同上 |
| #2 | 同上 |
| #4 | SU=('共病' OR '注意缺陷多动障碍' OR '多动症' OR 'ADHD' OR '强迫障碍' OR '强迫' OR 'OCD' OR '焦虑' OR '抑郁') |
| Final | #1 AND#2 AND #4 |

## A5-5d. Epidemiology / guidelines

| **Step** | **Search terms** |
| --- | --- |
| Final | (#1 AND#2) AND SU=('流行病学' OR '患病率' OR '发病率' OR '指南' OR '共识' OR '专家共识') |

## Time range

| **Category** | **Time range** |
| --- | --- |
| Intervention / efficacy / safety / comorbidity | 建库至 2025-05-31 |
| Epidemiology / guidelines | 2015-01-01 至 2025-05-31 |

# A5-6. SinoMed search strategy

## Field note

| **Field** | **Description** |
| --- | --- |
| MH | 主题词（CMeSH） |
| PY | 出版年字段 |

## A5-6a. Intervention / efficacy evidence

| **Step** | **Search terms** |
| --- | --- |
| #1 | (MH='抽动障碍' OR MH='妥瑞症' OR '抽动障碍' OR '妥瑞综合征' OR '抽动-秽语综合征' OR '抽动秽语综合征' OR '多发性抽动' OR '慢性抽动' OR '一过性抽动' OR '抽动症') |
| #2 | (MH='儿童' OR MH='青少年' OR '儿童' OR '青少年' OR '小儿' OR '学龄儿童' OR '未成年人' OR '儿科') |
| #3 | (MH='中西医结合疗法' OR MH='中医疗法' OR MH='针刺疗法' OR '中西医结合' OR '中西医' OR '中西医联合' OR '中西药' OR '中医治疗' OR '中医药' OR '中药' OR '中药方剂' OR '中成药' OR '中药制剂' OR '针刺' OR '针灸') |
| Final | #1 AND#2 AND#3 |

## A5-6b. Safety evidence

| **Step** | **Search terms** |
| --- | --- |
| #1 | 同上 |
| #2 | 同上 |
| #3 | 同上 |
| #4 | (MH='药物不良反应' OR '安全性' OR '不良反应' OR '不良事件' OR '耐受性') |
| Final | #1 AND#2 AND#3 AND #4 |

## A5-6c. Comorbidity evidence

| **Step** | **Search terms** |
| --- | --- |
| #1 | 同上 |
| #2 | 同上 |
| #4 | (MH='注意缺陷障碍伴多动' OR MH='强迫性障碍' OR MH='焦虑障碍' OR MH='抑郁障碍' OR '共病' OR '注意缺陷多动障碍' OR '多动症' OR 'ADHD' OR '强迫障碍' OR '强迫' OR 'OCD' OR '焦虑' OR '抑郁') |
| Final | #1 AND#2 AND #4 |

## A5-6d. Epidemiology / guidelines

| **Step** | **Search terms** |
| --- | --- |
| Final | (#1 AND#2) AND ('流行病学' OR '患病率' OR '发病率' OR '指南' OR '共识' OR '专家共识') AND (PY BETWEEN 2015 AND 2025) |

# A5-7. WanFang Data search strategy

## A5-7a. Intervention / efficacy evidence

| **Step** | **Search terms** |
| --- | --- |
| #1 | 主题:(抽动障碍 OR 妥瑞综合征 OR 抽动-秽语综合征 OR 抽动秽语综合征 OR 多发性抽动 OR 慢性抽动 OR 一过性抽动 OR 抽动症) |
| #2 | 主题:(儿童 OR 青少年 OR 小儿 OR 学龄儿童 OR 未成年人 OR 儿科) |
| #3 | 主题:(中西医结合 OR 中西医 OR 中西医联合 OR 中西药 OR 中医治疗 OR 中西医结合疗法 OR 中医药 OR 中药 OR 中药方剂 OR 中成药 OR 中药制剂 OR 针刺 OR 针灸) |
| Final | #1 AND#2 AND#3 |

## A5-7b. Safety evidence

| **Step** | **Search terms** |
| --- | --- |
| #1 | 同上 |
| #2 | 同上 |
| #3 | 同上 |
| #4 | 主题:(安全性 OR 不良反应 OR 不良事件 OR 耐受性) |
| Final | #1 AND#2 AND#3 AND #4 |

## A5-7c. Comorbidity evidence

| **Step** | **Search terms** |
| --- | --- |
| #1 | 同上 |
| #2 | 同上 |
| #4 | 主题:(共病 OR 注意缺陷多动障碍 OR 多动症 OR ADHD OR 强迫障碍 OR 强迫 OR OCD OR 焦虑 OR 抑郁) |
| Final | #1 AND#2 AND #4 |

## A5-7d. Epidemiology / guidelines

| **Step** | **Search terms** |
| --- | --- |
| Final | (#1 AND#2) AND 主题:(流行病学 OR 患病率 OR 发病率 OR 指南 OR 共识 OR 专家共识) |

## Time range

| **Category** | **Time range** |
| --- | --- |
| Intervention / efficacy / safety / comorbidity | 建库至 2025-05-31 |
| Epidemiology / guidelines | 2015-01-01 至 2025-05-31 |

# A5-8. VIP database search strategy

## A5-8a. Intervention / efficacy evidence

| **Step** | **Search terms** |
| --- | --- |
| #1 | U=(抽动障碍 OR 妥瑞综合征 OR 抽动-秽语综合征 OR 抽动秽语综合征 OR 多发性抽动 OR 慢性抽动 OR 一过性抽动 OR 抽动症) |
| #2 | U=(儿童 OR 青少年 OR 小儿 OR 学龄儿童 OR 未成年人 OR 儿科) |
| #3 | U=(中西医结合 OR 中西医 OR 中西医联合 OR 中西药 OR 中医治疗 OR 中西医结合疗法 OR 中医药 OR 中药 OR 中药方剂 OR 中成药 OR 中药制剂 OR 针刺 OR 针灸) |
| Final | #1 AND#2 AND#3 |

## A5-8b. Safety evidence

| **Step** | **Search terms** |
| --- | --- |
| #1 | 同上 |
| #2 | 同上 |
| #3 | 同上 |
| #4 | U=(安全性 OR 不良反应 OR 不良事件 OR 耐受性) |
| Final | #1 AND#2 AND#3 AND #4 |

## A5-8c. Comorbidity evidence

| **Step** | **Search terms** |
| --- | --- |
| #1 | 同上 |
| #2 | 同上 |
| #4 | U= (共病 OR 注意缺陷多动障碍 OR 多动症 OR ADHD OR 强迫障碍 OR 强迫 OR OCD OR 焦虑 OR 抑郁) |
| Final | #1 AND#2 AND #4 |

## A5-8d. Epidemiology / guidelines

| **Step** | **Search terms** |
| --- | --- |
| Final | (#1 AND#2) AND U=(流行病学 OR 患病率 OR 发病率 OR 指南 OR 共识 OR 专家共识) |

## Time range

| **Category** | **Time range** |
| --- | --- |
| Intervention / efficacy / safety / comorbidity | 建库至 2025-05-31 |
| Epidemiology / guidelines | 2015-01-01 至 2025-05-31 |

# A5-9. Trial registry searches

## A5-9. Registry search strategies

| **Registry** | **Search strategy** |
| --- | --- |
| ClinicalTrials.gov | Condition or disease: tic disorder OR Tourette syndrome; Population keywords: child OR adolescent OR pediatric; Intervention keywords where relevant: integrated medicine OR Chinese medicine OR traditional Chinese medicine OR Chinese herbal medicine OR acupuncture OR TCM |
| ChiCTR | 疾病名称：抽动障碍 OR 妥瑞综合征 OR 抽动-秽语综合征 OR 抽动症；人群：儿童 OR 小儿 OR 青少年；干预措施：中西医结合 OR 中医药 OR 中药 OR 针刺 OR 针灸；研究类型：以干预性研究为主 |
| WHO ICTRP | Basic search combinations included: tic disorder AND child; Tourette syndrome AND adolescent; traditional Chinese medicine AND tic disorder; acupuncture AND Tourette syndrome |

# A5-10. Grey literature search

## Sources searched

| **Source** | **Details** |
| --- | --- |
| National Health Commission of China | Guidelines, notices, and policy documents |
| China Association of Chinese Medicine | Consensus statements, expert documents, and society materials |
| World Health Organization | Global guideline and evidence documents |
| Google Scholar | Supplementary grey-literature search source |

## Example keyword combinations

| **Keyword combinations** |  |
| --- | --- |
| "tic disorder" AND child* AND guideline |  |
| "Tourette syndrome" AND pediatric AND consensus |  |
| "抽动障碍" AND 儿童 AND 指南 |  |
| "抽动障碍" AND 儿童 AND 共识 |  |
| "抽动障碍" AND 中西医结合 |  |

# Appendix 6: Foundational Data Extraction Form

## Recommendation-specific evidence tracing header

**Summary recommendation:** _______________________________________

**Transformed research question:** ___________________________________

**Associated clinical question / PICO domain:** ___________________________

Table A7-1. Tracing and Appraisal of Guideline Evidence

| Source guideline | Recommendation text | Referenced study | Study design | Level of support to recommendation* | Notes |
| --- | --- | --- | --- | --- | --- |
|  |  |  |  |  |  |
|  |  |  |  |  |  |
|  |  |  |  |  |  |

* **Level of support to recommendation:** 1 = strong/direct support; 2 = moderate/partial support; 3 = limited/indirect support; 4 = cited but not substantively supportive

Table A7-2. Study Characteristics and Data Extraction Template

| Field | Description / Data Entry |
| --- | --- |
| Study ID | First author, year |
| Full citation | Full bibliographic reference |
| Study design | RCT / quasi-experimental / cohort / case-control / cross-sectional / diagnostic accuracy / qualitative study / systematic review / meta-analysis / guideline |
| Follow-up duration | months/weeks |
| Setting | Country; institution type; inpatient/outpatient/community setting |
| Population | Age range; sex ratio; diagnostic criteria; TD subtype; comorbidity status |
| Sample size | Total sample; intervention group; comparator group |
| Intervention (I) | Name of formula/drug/intervention; dosage; frequency; duration; co-interventions |
| Comparator (C) | Western monotherapy / placebo / no treatment / behavioral intervention / usual care / other |
| TCM syndrome pattern | liver‑wind stirring / spleen deficiency with liver hyperactivity / yin deficiency with wind agitation / phlegm‑fire disturbing spirit / external wind triggering / other |
| Primary outcome(s) | YGTSS total score; tic severity; predefined primary endpoint(s) |
| Secondary outcome(s) | TCM syndrome score; SNAP-IV; Y-BOCS; RCADS-25; GTS-QOL; functional outcomes; HRV-SDNN; other |
| Safety outcomes | Adverse events; severity grade; discontinuation; incidence rate |
| Effect estimate | RR / OR / MD / SMD with 95% CI; P value; adjusted estimate if applicable |
| Key findings | Brief summary of main efficacy and/or safety results |
| Patient and family perspectives | Qualitative findings or patient-reported outcomes, if available |
| Conflict of interest | Declared / Not declared / Unclear |
| Study quality / risk of bias | Tool used; overall rating; domain-specific judgments |
| Reviewer notes | Extraction discrepancies; assumptions; additional observations |

# Appendix 7: GRADE Evidence Assessment Table

## Table A7-1. Outcome-level GRADE evidence profile

Clinical question (PICO-formulated):_________________________________________________

| No. of studies | Study design | Risk of bias | Inconsistency | Indirectness | Imprecision | Other considerations | Relative effect | Absolute effect | Certainty | Importance | Comments |
| --- | --- | --- | --- | --- | --- | --- | --- | --- | --- | --- | --- |
| 1 |  |  |  |  |  |  |  |  |  |  |  |
| 2 |  |  |  |  |  |  |  |  |  |  |  |
| … |  |  |  |  |  |  |  |  |  |  |  |

| **Table A7-3. GRADE Evidence Assessment Table** | | | |
| --- | --- | --- | --- |
| Domain | Assessment Criteria | Judgment | Rationale / Explanation |
| Downgrading Factors | | | |
| 1. Risk of Bias | Limitations in study design, execution, or analysis (randomization, blinding, attrition, selective reporting) | [Not serious / Serious / Very serious] | [e.g., 3 of 5 RCTs had unclear allocation concealment; downgraded by 1 level] |
| 2. Inconsistency | Unexplained heterogeneity in results across studies (I², direction of effects) | [Not serious / Serious / Very serious] | [e.g., I² = 72%, effects inconsistent across studies; downgraded by 1 level] |
| 3. Indirectness | Differences in population, intervention, comparator, or outcomes vs. the clinical question | [Not serious / Serious / Very serious] | [e.g., Most studies used adult populations; downgraded by 1 level] |
| 4. Imprecision | Wide confidence intervals, small sample sizes, few events | [Not serious / Serious / Very serious] | [e.g., Total n < 100, 95% CI crosses null; downgraded by 1 level] |
| 5. Publication Bias | Evidence of selective publication (funnel plot asymmetry, Egger test) | [Undetected / Strongly suspected] | [e.g., Funnel plot symmetric; no downgrade] |
| Upgrading Factors | | | |
| 6. Large Effect | Effect size is large enough to be unlikely due to bias alone (RR > 2 or < 0.5) | [No / Yes] | [e.g., RR = 0.35, consistent across studies; upgraded by 1 level] |
| 7. Dose-Response | Clear dose-response gradient across studies | [No / Yes] | [e.g., Higher TCM dose associated with greater symptom reduction] |
| 8. Residual Confounding | All plausible confounders would reduce the observed effect, increasing confidence | [No / Yes] | [e.g., No residual confounders identified] |
|  | | | |
| FINAL CERTAINTY |  | High/Moderate /Low/Very Low | [Summary: Started as [X], downgraded [N] levels for [domains], upgraded [N] levels for [domains]. Final: [Grade]] |

Note: This template is repeated for each of the 10 core clinical questions. A = High; B = Moderate; C = Low; D = Very Low.

Table A7-3. Evidence-to-Decision framework

| Criteria | Judgments | Research evidence | Comments |
| --- | --- | --- | --- |
| Problem | No / Probably no / Uncertain / Probably yes / Yes / Varies |  |  |
| Certainty of evidence | Very low / Low / Moderate / High / No included studies |  |  |
| Desirable effects | No / Probably no / Uncertain / Probably yes / Yes / Varies |  |  |
| Undesirable effects | No / Probably no / Uncertain / Probably yes / Yes / Varies |  |  |
| Values | Important uncertainty / Possibly important uncertainty / Probably no important uncertainty / No important uncertainty |  |  |
| Balance of effects | Favors comparison / Probably favors comparison / Neither / Probably favors intervention / Favors intervention / Varies |  |  |
| Resources required | No / Probably no / Uncertain / Probably yes / Yes / Varies |  |  |
| Equity | Increased / Probably increased / Uncertain / Probably reduced / Reduced / Varies |  |  |
| Acceptability | No / Probably no / Uncertain / Probably yes / Yes / Varies |  |  |
| Feasibility | No / Probably no / Uncertain / Probably yes / Yes / Varies |  |  |

Table A7-4. Summary judgments

| Criterion | Summary judgment | Rationale (brief) |
| --- | --- | --- |
| Problem |  |  |
| Certainty of evidence |  |  |
| Desirable effects |  |  |
| Undesirable effects |  |  |
| Values |  |  |
| Balance of effects |  |  |
| Resources required |  |  |
| Equity |  |  |
| Acceptability |  |  |
| Feasibility |  |  |
| Recommendation direction | For / Against |  |
| Recommendation strength | Strong / Weak / Good Practice Statement / No consensus |  |

*For Good Practice Statements (GPS), Tables A8‑1 to A8‑3 are not applicable. Only Table A8‑4 is completed, with “GPS” selected as recommendation strength and the justification documented.*

# Appendix 8: Delphi Questionnaire Sample

Modified Delphi Survey — Round [1/2/3]

Dear Expert,

Thank you for participating in this modified Delphi survey. Please review the evidence summary and draft recommendation for each clinical question, and provide your rating and comments. Your responses will remain anonymous throughout the process.

**Rating scale:** 1 = strongly disagree; 2 = disagree; 3 = mostly disagree; 4 = somewhat disagree; 5 = uncertain; 6 = somewhat agree; 7 = mostly agree; 8 = agree; 9 = strongly agree.

Consensus threshold: ≥75% of panelists scoring 4 or 5.If you assign a score of ≤3, please provide your rationale in the comments field.

| Clinical Question Q1 |  |
| --- | --- |
| Evidence Summary |  |
| GRADE Certainty |  |
| Draft Recommendation |  |
| Recommendation Strength |  |

| Your Rating | 1 □ 2 □ 3 □ 4 □ 5 □ 6 □ 7 □ 8 □ 9 □ |
| --- | --- |
| Comments (required if score ≤6) |  |
| Suggested modifications (if any) |  |
| Round-specific feedback from previous round (if applicable) |  |

# Appendix 9: Expert Engagement and Concordance Coefficients

## 1. Response rate (panel engagement)

The response rate reflects expert participation in each Delphi round and was calculated as: **Response rate (%) = (number of completed questionnaires / number of distributed questionnaires) × 100.**

A response rate of **≥70%** was considered acceptable for continuation of the Delphi process.

| Round | Distributed (n) | Completed (n) | Response rate (%) |
| --- | --- | --- | --- |
| Round 1 |  |  |  |
| Round 2 |  |  |  |
| Round 3 |  |  |  |

**2. Coefficient of Variation (CV)**

The CV measures the degree of response concentration at the item level: CV = (Standard Deviation / Mean) × 100%.

Lower CV values indicate greater concentration of ratings, whereas higher CV values suggest more dispersed opinions and may warrant further discussion in conjunction with the predefined agreement threshold.

**3. Kendall’s Coefficient of Concordance (W)**

Kendall’s W assesses the overall consistency of expert rankings across all items within a round: W = 12S / [k² (n³ – n)].

Where: S = sum of squares of rank sums; k = number of experts; n = number of items rated.

W ranges from 0 (no agreement) to 1 (complete agreement). W > 0.5 with p < 0.05 indicates acceptable concordance.

| Round | Kendall’s W | Chi-square (χ²) | df | p |
| --- | --- | --- | --- | --- |
| Round 1 |  |  |  |  |
| Round 2 |  |  |  |  |
| Round 3 |  |  |  |  |

**4. Item-Level Consensus Summary (per round)**

The following statistics were calculated for each item in each Delphi round: median, interquartile range (IQR), mean, standard deviation (SD), coefficient of variation (CV), and the proportion of panelists scoring **7–9** on the 9-point Likert scale. Items reaching the prespecified agreement threshold (**≥75% of panelists scoring 7–9**) were classified as agreed and removed from subsequent rounds unless substantial wording revision was required.

| Item | Median | IQR | Mean ± SD | CV (%) | Agreement [(n)%] | Consensus Reached (Yes/No) |
| --- | --- | --- | --- | --- | --- | --- |
| Q1 |  |  |  |  |  |  |
| Q2 |  |  |  |  |  |  |
| Q3 |  |  |  |  |  |  |
| ... |  |  |  |  |  |  |

Note: This table should be reported separately for each Delphi round.

# Appendix 10: Structured External Review Form

**A. Reviewer information and conflict-of-interest declaration**

| Reviewer name |  | Date of review |  |
| --- | --- | --- | --- |
| Institution |  | Country/region |  |
| Primary expertise |  | Professional role/title |  |
| Relevant fields | Pediatric tic disorders / pediatric neurology / child psychiatry / psychology / integrative medicine / traditional Chinese medicine pediatrics / evidence-based medicine / guideline methodology / other: ________ | Previous involvement in this project | [ ] No  [ ] Yes, specify: __________ |
| Conflict of interest declaration | [ ] No relevant conflict of interest  [ ] Potential conflict of interest disclosed below | COI details |  |
| Permission to acknowledge reviewer | [ ] Yes  [ ] No | Confidential comments to editors/Steering Committee |  |
| Reviewer signature |  | Email |  |

**B. Domain-based external appraisal**

Please complete all relevant items. Use the comments column to identify whether a concern requires no change, editorial revision, substantive revision, or further panel consultation.

Rating scale: 1 = strongly disagree; 2 = disagree; 3 = mostly disagree; 4 = somewhat disagree; 5 = uncertain; 6 = somewhat agree; 7 = mostly agree; 8 = agree; 9 = strongly agree.

| No. | Domain | Appraisal item | Rating (1–9 / N/A) | Comments and suggested revisions |
| --- | --- | --- | --- | --- |
| 1 | Scope and target population | The scope of the consensus is clearly defined, including the target population of children and adolescents with tic disorders. |  |  |
| 2 | Scope and target population | The intended users of the consensus are clearly specified and appropriate for pediatric tic disorder care. |  |  |
| 3 | Scope and target population | The clinical questions and PICO framework are aligned with the stated scope and intended outputs. |  |  |
| 4 | Clinical relevance | The consensus addresses important clinical problems in pediatric tic disorder assessment and management. |  |  |
| 5 | Clinical relevance | The proposed recommendations are likely to be useful for frontline clinicians in routine practice. |  |  |
| 6 | Clinical relevance | The protocol appropriately considers functional impairment, school and social functioning, family burden, and quality of life in addition to tic severity. |  |  |
| 7 | Comorbidity coverage | Major comorbidities, including attention-deficit/hyperactivity disorder, obsessive-compulsive disorder, anxiety, and depressive symptoms, are adequately addressed. |  |  |
| 8 | Comorbidity coverage | The approach to comorbidity management is clinically coherent and sufficiently integrated with tic disorder management. |  |  |
| 9 | Outcome assessment | The efficacy evaluation framework is sufficiently multidimensional, including tic severity, traditional Chinese medicine syndrome patterns, functional outcomes, quality of life, and safety. |  |  |
| 10 | Outcome assessment | The proposed outcome domains are measurable, interpretable, and appropriate for pediatric clinical practice. |  |  |
| 11 | Evidence summaries | Evidence summaries are accurate, balanced, and sufficiently concise for decision-making. |  |  |
| 12 | Evidence summaries | The relationship between evidence summaries and draft recommendations is transparent. |  |  |
| 13 | GRADE certainty | The certainty of evidence is assessed and presented appropriately using GRADE or other suitable frameworks where applicable. |  |  |
| 14 | Evidence-to-Decision | The Evidence-to-Decision considerations adequately address benefits, harms, patient values and preferences, resources, equity, acceptability, and feasibility. |  |  |
| 15 | Recommendation wording | Recommendation statements are clear, actionable, and unambiguous. |  |  |
| 16 | Recommendation wording | The direction and strength of each recommendation are consistent with the evidence certainty and Evidence-to-Decision judgments. |  |  |
| 17 | Good Practice Statements | Good Practice Statements, if used, are clearly distinguished from evidence-based recommendations and are justified appropriately. |  |  |
| 18 | Safety monitoring | Safety monitoring and adverse-event management principles are sufficiently described. |  |  |
| 19 | Safety monitoring | Potential safety concerns related to integrative treatment approaches are adequately considered. |  |  |
| 20 | Feasibility and acceptability | The recommendations are feasible and acceptable in the anticipated clinical settings. |  |  |
| 21 | Feasibility and acceptability | Resource requirements and implementation barriers are adequately considered. |  |  |
| 22 | Equity considerations | The consensus adequately considers equity, accessibility, and variability across regions and clinical settings. |  |  |
| 23 | Methodological transparency | The Delphi process, voting thresholds, conflict-of-interest management, and consensus meeting rules are sufficiently transparent. |  |  |
| 24 | Methodological transparency | The external review and final approval process are clearly described and appropriate. |  |  |
| 25 | Implementation and updating | The dissemination and updating plan is appropriate and likely to maintain the relevance of the consensus. |  |  |
| 26 | Overall quality | Overall, the document is methodologically robust, clinically useful, and suitable for endorsement after appropriate revision. |  |  |

**C. Recommendation-by-recommendation review**

Please use this table for specific comments on individual recommendations. Add rows as needed. “Suggested action” may be: no change, editorial revision, substantive revision, further panel consultation, or exclusion.

| Recommendation ID | Recommendation statement / section | Clarity | Evidence alignment | Feasibility | Suggested action | Specific comment / proposed wording |
| --- | --- | --- | --- | --- | --- | --- |
| R1 |  |  |  |  |  |  |
| R2 |  |  |  |  |  |  |
| … |  |  |  |  |  |  |

**D. Summary of major issues requiring Steering Committee response**

| No. | Section / recommendation | Major issue | Suggested revision | Priority (High/Moderate/Low) |
| --- | --- | --- | --- | --- |
| 1 |  |  |  |  |
| 2 |  |  |  |  |
| … |  |  |  |  |

**E. Overall recommendation**

| Overall endorsement decision | [ ] Endorse without revision [ ] Endorse with minor revision [ ] Reconsider after major revision [ ] Do not endorse |
| --- | --- |
| Overall confidence in the consensus document | [ ] Very low [ ] Low [ ] Moderate [ ] High [ ] Very high |
| Most important strengths |  |
| Most important limitations |  |
| Additional comments for the Steering Committee |  |

**F. Steering Committee response log (to be completed by the Steering Committee)**

The following table is provided to ensure a transparent audit trail for external review comments.

| No. | Reviewer comment | Steering Committee response | Decision | Revision made | Need panel verification? | Date completed |
| --- | --- | --- | --- | --- | --- | --- |
| 1 |  |  |  |  |  |  |
| 2 |  |  |  |  |  |  |
| 3 |  |  |  |  |  |  |
| 4 |  |  |  |  |  |  |
| 5 |  |  |  |  |  |  |
| 6 |  |  |  |  |  |  |
| 7 |  |  |  |  |  |  |
| 8 |  |  |  |  |  |  |

**Reviewer declaration: I confirm that I have completed this external review independently and have disclosed any potential conflicts of interest relevant to this consensus document.**

| **Signature** |  | **Date** |  |
| --- | --- | --- | --- |
